# Prevalence and multidrug resistance of *Enterococcus* species isolated from chickens at slaughterhouses in Nakhon Ratchasima Province, Thailand

**DOI:** 10.14202/vetworld.2022.2535-2542

**Published:** 2022-11-10

**Authors:** Pattarakitti Noenchat, Chawakorn Nhoonoi, Thanawan Srithong, Sujeeporn Lertpiriyasakulkit, Pairat Sornplang

**Affiliations:** 1 Sakon Nakhon Provincial Livestock Office, Department of Livestock Development, Ministry of Agriculture and Cooperatives, Sakon Nakhon 47000, Thailand; 2 Division of Veterinary Public Health, Faculty of Veterinary Medicine, Khon Kaen University, Khon Kaen 40002, Thailand

**Keywords:** antimicrobial agents, antimicrobial resistance, *Enterococcus* species, multidrug resistance, poultry

## Abstract

**Background and Aim:**

*Enterococcus* is a commensal bacteria found in humans and animals, which can cause human nosocomial infections. One of the most contaminated enterococcal sources is poultry meat. Therefore, this study estimated the prevalence and antimicrobial resistance (AMR) profile of *Enterococcus* from chickens and their meat products at local slaughterhouses in Nakhon Ratchasima Province, Thailand.

**Materials and Methods:**

From January 2021 to March 2022, 558 samples from 279 cloacal swabs and breast meat were collected from 31 local slaughterhouses in the area. Then, the samples were screened for *Enterococcus* using modified de Man, Rogosa, and Sharpe agar. Next, selected Gram-positive, catalase-negative, and cocci-shaped colonies were investigated for enterococcal confirmation using Enterococcosel Agar (EA). We also cultivated the samples directly on EA. However, the disk diffusion method was used to investigate positive *Enterococcus* resistance profiles to 16 antimicrobial agents. Finally, selected phenotypic multidrug-resistant (MDR) *Enterococcus* isolates were further assessed to identify AMR genes by polymerase chain reaction.

**Results:**

Investigations showed that the prevalence of *Enterococcus* isolates from the chicken cloacal swabs and meat samples were 29.75% (83/279) and 28.32% (78/279), respectively. Most *Enterococcus* positive isolates were resistant to colistin, followed by cefoxitin, cephalexin, and streptomycin. These isolates also showed a prevalence of MDR species (65.22%; 105/161) and 66 patterns. Furthermore, selected MDR *Enterococcus* (MDRE) from cloacal swabs and breast meat were positive for the resistant extended**-**spectrum beta-lactamase TEM genes at 71.43% (20/28) and 78.26% (18/23), respectively, whereas other AMR genes detected in the selected MDR enterococci from the cloacal swabs and breast meat were beta-lactamase TEM (*bla*_TEM_ [0%, 1.96%]), Class 1 integrase (*intI1* [14.28%, 0%]), colistin (*mrc–1* [3.57%, 0%]), and vancomycin (*vanA* [14.28%, 0%]).

**Conclusion:**

This study indicated that phenotypic MDRE correlated with extended-spectrum beta-lactamase TEM gene presence, leading to an AMR reservoir that can be transferred to other bacteria.

## Introduction

Poultry meat is popularly consumed and easily distributed in the Thai market. Hence, Thailand has become the fourth country in distributing processed chicken meat to world chicken exports in 2022. Frozen meat has also been sixth-ranked among world chicken exporters [1]. Specifically, Nakhon Ratchasima Province is one of the three provinces with the highest broiler production in Thailand.

Poultry and poultry products have been proposed as a primary source of bacterial contamination, especially from the poultry farms or during slaughtering, causing human foodborne illness through consumed meat. Additionally, antibiotic-resistant bacteria could remain in the human body through food, posing a public health concern worldwide because of their problematic treatment. Besides, previous Thai studies have reported the presence of antibiotic-resistant phenotypic and genotypic *Enterobacteriaceae* isolates in chicken feces and meat. However, *Enterococcus* isolates’ prevalence, including their phenotypic and genotypic antimicrobial resistance (AMR) remains unknown.

Multidrug-resistant (MDR) bacteria, which are resistant to at least three antimicrobial classes, lead to more AMR bacteria outbreaks worldwide. Most AMR genes (AMRGs) and genetic elements found in pathogenic *Enterobacteriaceae* are extended-spectrum beta-lactamase (ESBL), beta-lactamase (*bla*), Class 1 integrase (*intI1*), and colistin (CT; *mcr-1*). Although the ESBL-encoding gene, particularly TEM, has frequently been detected in chickens and chicken meat worldwide [2], Class 1 integron has been identified as the most common resistance integron. This integron comprises gene cassettes of distributed AMR in most Gram-negative enteric bacteria, which majorly influences the antimicrobial and MDR nature of many bacterial species [3]. However, these genes have not been fully addressed in commensal bacteria.

*Enterococcus* species (*Enterococcus* spp.) are commensal and opportunistic pathogenic bacteria that serve as probiotics in humans and animals. For example, vancomycin (VA)-resistant enterococci (VRE) causes nosocomial infections, such as urinary tract infections, wound infections, and endocarditis [4]. The three most common variants of VA resistance genes reported in *Enterococcus* are *vanA*, *vanB*, and *vanC* [5, 6]. Furthermore, *Enterococcus* has a high probability of exposure to many antibiotics, making it an indicator bacterium widely used for AMR monitoring [7].

Therefore, this study aimed to investigate the prevalence and AMR profile of *Enterococcus* isolated from chickens and their meat products at local slaughterhouses in Nakhon Ratchasima Province, Thailand.

## Materials and Methods

### Ethical approval

The use of animals in this study was approved under the permissions and guidelines of the Institutional Animal Care and Use Committee of Khon Kaen University, Thailand (permission record no. IACUC-KKU-79/64).

### Study period and location

The study was conducted from January 2021 to March 2022. Nine cloacal swabs and breast meat samples were randomly collected from each of the 31 local poultry slaughterhouses from 50 chicken farms in 12 districts in Nakhon Ratchasima Province, Thailand, using a cross-sectional design. All the farms and slaughterhouses have been certified to raise and slaughter inland by the Thai government. A total of 558 samples were collected (279 from cloacal swabs and breast meat) from various chicken types, comprising broilers (432 samples), indigenous chickens (90 samples), and spent laying hens (36 samples). The samples (pre-and post-slaughtered chicken) were kept at 4°C and transferred to a microbiology laboratory at the Department of Veterinary Public Health, Faculty of Veterinary Medicine, Khon Kaen University, Thailand, within 24 h, for microbiological investigations.

### *Enterococcus* isolation

Based on the two groups of *Enterococcus* strains present in the samples, the non-pathogenic lactic acid-producing *Enterococcus* and the potential pathogenic *Enterococcus*, each sample was cultivated on different selective media. For non-pathogenic *Enterococcus* isolation, the samples were grown using lactic acid bacteria isolation, according to Sornplang *et al*. [8]. Briefly, all cloacal swab samples (approximately 1 g of intestinal contents) were first placed in a sterile tube to prepare a ten-fold serial dilution (to 10^−4^ concentration) using 10 of mL buffered peptone water (BPW; Oxoid, Hampshire, UK). Then, 25 g of chicken breast was diluted with 225 mL of BPW and mixed using a stomacher for 3 min at 25°C, after which the sample was subjected to a ten-fold serial dilution (to a 10^−4^ concentration) with BPW. Subsequently, a 1 mL aliquot of the 10^-4^ dilution was pipetted on sterile plates. Then, de Man, Rogosa, and Sharpe agar (Difco, USA), with the modification of adding 0.4 % (w/v) CaCO_3_ was poured onto the plates. Finally, the Petri plates were incubated in 5% CO_2_ for 48 h at 37°C. One selected positive colony of *Enterococcus* spp. from each collected sample was confirmed through Gram staining and catalase test. The same prepared samples of lactic acid-producing *Enterococcus* were cultivated on Enterococcosel Agar (BD, Germany) for potential pathogenic *Enterococcus* isolation. Alternatively, *Enterococcus faecalis* (ATCC 29212), which grew on the medium as beige-colored, intense-black halo colonies, was used as a positive control [9]. The positive criteria of both *Enterococcus* groups were selected for the antimicrobial susceptibility test.

### Antimicrobial susceptibility test

The *Enterococcus*-positive samples were tested for antimicrobial susceptibility using the Kirby–Bauer disk diffusion method [10]. Then, the antimicrobial susceptibility of the *Enterococcus* isolates was interpreted using the Clinical and Laboratory Standards Institute guidelines [11]. Subsequently, we chose 16 antimicrobial agents with various antibacterial activities in eight different antibiotic classes, commonly used to treat infections in humans and animals, including aminoglycoside (neomycin [N, 30 μg] and streptomycin [S, 10 μg]), beta-lactam (cephalosporin and penicillin) (cephalexin [CL, 30 μg], cefepime [FEP, 30 μg], cefotaxime [CTX, 30 μg], cefoxitin [FOX, 30 μg], amoxicillin [AML, 10 μg], and ampicillin [AMP, 10 μg]), chloramphenicol (C, 30 μg), fluoroquinolone (enrofloxacin [ENR, 5 μg]), macrolide (erythromycin [E, 15 μg]), peptide (CT, [10 μg], and VA, [30 μg]), sulfonamide (sulfamethoxazole/trimethoprim [19:1] [SXT, 25 μg]), and tetracycline classes (doxycycline [DO, 30 μg] and oxytetracycline [OT, 30 μg]), for investigations. All antimicrobial disks were obtained from Oxoid (Oxoid, UK).

### Preparation of bacterial inoculum

A selected *Enterococcus* colony was suspended in 2 mL 0.85% (w/v) of normal saline. Then, the inoculum was adjusted to a turbidity equivalent to that of a 0.5 McFarland standard (1.5 × 10^8^ CFU/mL). Next, the inoculum was dipped using a sterile cotton swab and swabbed onto the Mueller-Hinton agar (Becton Dickinson, USA) plate, followed by inoculation of plates and incubation under aerobic conditions at 5% CO_2_, and 37°C for 48 h. *Escherichia coli* ATCC25922 was used as the reference strain.

### Primers used and polymerase chain reaction (PCR) conditions

Phenotypic MDRE isolates were further investigated for the presence of five AMRGs: ESBL-TEM, *bla*_TEM_, *intI1*, *mrc-1*, and *vanA*. These genes were commonly found in pathogenic bacteria. DNA was extracted and purified from the overnight culture of the positive phenotypic MDRE isolates using a FastGene Gel/PCR extraction kit (Nippon, Germany) as per the manufacturer’s instructions. The polymerase chain reaction was conducted in 25 mL of the reaction mixture containing bacterial cells as the source of DNA template, Taq DNA polymerase (Merk, Germany), PCR grade water (Thermo Scientific), and specific primer pairs (Thermo Scientific). Thermocycling followed the PCR conditions of the gene primers used, which are listed in Table-1 [12–14]. The 2% agarose gel was prepared and electrophoresed with 0.5 M Tris-Borate-EDTA buffer. Electrophoresis was performed at 76 V (constant voltage) for approximately 30 min. The gels were stained with an ethidium bromide solution (5 mg/mL) for 20 min, washed with deionized water, and viewed by ultraviolet transillumination.

**Table-1:** The five gene specific primers used in this study.

Gene	Sequence (5’–3’) (F=forward, R=reverse)	Annealing temperature (°C)	Amplicon size (bp)	Reference
ESBL–TEM	F-TTTCGTGTCGCCCTTATTCC	50	404	[12]
ESBL–TEM	R-ATCGTTGTCAGAAGTAAGTTGG	50	404	[12]
*bla*_TEM_	F-CATTTCCGTGTCGCCCTTAT	55	793	[12]
*bla*_TEM_	R-TCCATAGTTGCCTGACTCCC	55	793	[12]
*intI1*	F-GGGTCAAGGATCTGGATTTCG	62	483	[13]
*intI1*	R-ACATGGGTGTAAATCATCGTC	62	483	[13]
*mrc–1*	F-AGTCCGTTTGTTCTTGTGGC	58	320	[13]
*mrc–1*	R-AGATCCTTGGTCTCGGCTTG	58	320	[13]
*vanA*	F-GGGAAAACGACAATTGC	54	723	[14]
*vanA*	R-GTACAATGCGGCCGTTA	57	723	[14]

ESBL–TEM=Extended-spectrum beta-lactamase–TEM

### Statistical analysis

The MDR pattern is described as resistance to antibiotics in a minimum of three antimicrobial classes. First, positive *Enterococcus* isolates from chicken cloacal swabs, chicken breast meat, and phenotypic/genotypic AMR profiles were reported in percentages. Then, the Chi-square test was used to test the relationship between the diverse prevalence of chicken cloacal swabs and chicken breast meat samples in *Enterococcus* isolates and their phenotypic/genotypic AMR profiles. Finally, statistical analyses were performed using SPSS (v. 16.0; SPSS Inc., Chicago, IL, USA), and we considered the significant difference at p < 0.05.

## Results

### Prevalence of the *Enterococcus* spp.

Of the 558 samples investigated, 161 were *Enterococcus* positive. The prevalence rates of *Enterococcus* isolates from the cloacal swab and meat samples were 29.75% (83/279) and 27.95% (78/279), respectively. However, this prevalence rate was not significantly different (p > 0.05).

### Phenotypic AMR profiles of the *Enterococcus* spp.

Most of the *Enterococcus* spp. isolated from the cloacal swabs and breast meat samples were resistant to CL (81.93%, 83.33%), CT (98.80%, 97.44%), FOX (83.13%, 85.90%), and S (65.06%, 55.13%). Specifically, all *Enterococcus* spp. isolated from chicken breast meat were sensitive to AMP, and some were sensitive to nine antimicrobial agents (AML, C, VA, ENR, N, SXT, DO, OT, and E) at prevalence rates of 88.46%–98.72% (Table-2). In contrast, the AMR prevalence rate of *Enterococcus* spp. isolated from cloacal swabs, which were resistant to five antibiotics (CTX, E, ENR, FEP, and S), was significantly higher than those isolated from the breast meat (p < 0.05).

Of the 161 isolates, 105 were MDRE. We also observed that the MDRE from chicken cloacal swabs (61/105) was significantly higher than that from chicken breast meat (44/105) (p = 0.023), it was resistant to 3–12 antimicrobial agents in 3–7 antimicrobial classes. Moreover, the MDRE pattern of *Enterococcus* isolates from cloacal swabs and breast meat were 44 and 22, respectively (Table-3).

**Table-2:** Phenotypic antimicrobial-resistant *Enterococcus* isolated from chickens at local slaughterhouses in Nakhon Ratchasima Province, Thailand.

Sample	Percentage of AMR^1^ to antimicrobial agents (no. of isolates)							

	AML	AMP	C	CL	CT	CTX	DO	E
Cloacal swab	7.23 (6/83)	2.41 (2/83)	1.20 (1/83)	81.93 (68/83)	98.80 (82/83)	37.35 (31/83)	6.02 (5/83)	18.07 (15/83)
Breast meat	1.28 (1/78)	0 (0)	1.28 (1/78)	83.33 (65/78)	97.44 (76/78)	21.79 (17/78)	6.41 (5/78)	5.13 (4/78)
p*-*value	0.118	-	1.000	0.814	0.611	0.031*	1.000	0.011*

**Sample**	**Percentage of AMR^1^ to antimicrobial agents (no. of isolates)**							

	**ENR**	**FEP**	**FOX**	**N**	**OT**	**S**	**SXT**	**VA**

Cloacal swab	12.05 (10/83)	39.76 (33/83)	83.13 (69/83)	14.46 (12/83)	33.73 (28/83)	65.06 (54/83)	15.66 (13/83)	15.66 (13/83)
Breast meat	2.56 (2/78)	24.36 (19/78)	85.90 (67/78)	7.69 (6/78)	11.54 (9/78)	55.13 (43/78)	6.41 (5/78)	7.69 (6/78)
p*-*value	0.022*	0.022*	0.628	0.215	0.001*	0.198	0.081	0.145

AML=Amoxicillin, AMP=Ampicillin, C=Chloramphenicol, CL=Cephalexin, CT=Colistin, CTX=Cefotaxime, DO=Doxycycline, E=Erythromycin, ENR=Enrofloxacin, FEP=Cefepime, FOX=Cefoxitin, N=Neomycin, OT=Oxytetracycline, S=Streptomycin, SXT=Sulfamethoxazole+trimethoprim, VA=Vancomycin. ^1^Antimicrobial resistance.

* The AMR profile between cloacal swabs and breast meat samples was significantly different (p < 0.05).

**Table-3:** Chicken cloacal swabs (CS) and Breast meat (BM) AMR^1^-*Enterococcus* profiles from local slaughterhouses in Nakhon Ratchasima Province, Thailand.

Sample	No. of AM^2^	No. of AM classes	Resistance phenotypic profiles	No. of isolates (%)	p-value^3^
BM	2	1	CL-FOX	1 (0.62) (ND^4^ in CS)	0.485
CS	2	2	CT-FOX	1 (0.62) (ND in BM)	0.132
BM	2	2	CT-S	3 (1.86) (ND in CS)	0.170
BM	3	2	CL-CT-CTX	1 (0.62) (ND in CS)	0.485
CS	3	2	CT-CTX-FOX	1 (0.62) (ND in BM)	0.132
CSBM	3	2	CL-CT-FOX	14 (8.69)	0.553
	3	2	CL-CT-FOX	18 (11.18)	
CS	3	2	CT-FEP-FOX	1 (0.62)	0.485
BM	3	2	CT-FEP-FOX	1 (0.62)	
CS	4	2	AML-CL-CT-FOX	1 (0.62) (ND in BM)	0.132
CSBM	4	2	CL-CT-CTX-FOX	1 (0.62)	0.485
	4	2	CL-CT-CTX-FOX	1 (0.62)	
CSBM	4	2	CL-CT-FEP-FOX	2 (1.24)	0.025*
	4	2	CL-CT-FEP-FOX	5 (3.10)	
BM	4	2	CL-CT-FOX-VA	1 (0.62) (ND in CS)	0.485
CSBM	5	2	CL-CT-CTX-FEP-FOX	1 (0.62)	0.611
	5	2	CL-CT-CTX-FEP-FOX	2 (1.24)	
CS	3	3	CL-SXT-VA	1 (0.62) (ND in BM)	0.132
CS	3	3	CT-E-S	1 (0.62) (ND in BM)	0.132
CS	3	3	CT-FOX-S	4 (2.48)	0.682
BM	3	3	CT-FOX-S	2 (1.24)	
CSBM	4	3	CL-CT-FOX-S	9 (5.59)	0.024*
	4	3	CL-CT-FOX-S	19 (11.80)	
CS	4	3	CL-CT-FOX-OT	1 (0.62) (ND in BM)	0.132
CS	4	3	CL-CT-CTX -N	1 (0.62) (ND in BM)	0.132
BM	4	3	CL-CT-CTX-DO	1 (0.62) (ND in CS)	0.485
BM	4	3	CL-CT-DO-OT	1 (0.62) (ND in CS)	0.485
BM	4	3	CT-CTX-FOX-OT	1 (0.62) (ND in CS)	0.485
CS	4	3	CT-FEP-FOX-S	1 (0.62) (ND in BM)	0.132
BM	5	3	CL-CT-CTX-FOX-N	1 (0.62) (ND in CS)	0.485
CS	5	3	CL-CT-CTX-FOX-S	1 (0.62) (ND in BM)	0.132
BM	5	3	CL-CT-CTX-FOX-SXT	1 (0.62) (ND in CS)	0.485
CS	5	3	CL-CT-FEP-FOX-S	3 (1.86) (ND in BM)	0.034*
CS	5	3	CL-CT-CTX-FOX-OT	1 (0.62) (ND in BM)	0.132
CS	6	3	CL-CT-CTX-FEP-FOX-S	1 (0.62) (ND in BM)	0.132
BM	6	3	FEP-FOX-CT-N-S-VA	1 (0.62) (ND in CS)	0.485
CS	7	3	CL-CT-CTX-FEP-FOX-SXT-VA	1 (0.62) (ND in BM)	0.132
CS	7	3	CL-CT-FEP-FOX-N-S-VA	1 (0.62) (ND in BM)	0.132
CSBM	8	3	CL-CT-CTX-FOX-FEP-N-S-VA	2 (1.24)	0.476
	8	3	CL-CT-CTX-FOX-FEP-N-S-VA	2 (1.24)	
CS	4	4	CT-FOX-OT-S	2 (1.24) (ND in BM)	0.068
BM	4	4	CT-E-FOX-S	1 (0.62) (ND in CS)	0.485
BM	4	4	CT-CTX-DO-S	1 (0.62) (ND in CS)	0.485
BM	4	4	CT- E-OT-S	1 (0.62) (ND in CS)	0.485
BM	5	4	CL-CT-FOX-S-SXT	1 (0.62) (ND in CS)	0.485
CS	5	4	CL-CT-CTX-OT-S	1 (0.62) (ND in BM)	0.132
CSBM	5	4	CL-CT-FOX-OT-S	1 (0.62)	0.485
	5	4	CL-CT-FOX-OT-S	1 (0.62)	
CS	5	4	CT- DO-FOX-S-OT	1 (0.62) (ND in BM)	0.132
BM	5	4	CT-CTX-FEP-S-SXT	2 (1.24) (ND in CS)	0.294
BM	6	4	CL-CT-FEP-FOX-S-SXT	1 (0.62) (ND in CS)	0.485
CS	6	4	CL-CT-CTX-FEP-S-SXT	1 (0.62) (ND in BM)	0.132
BM	6	4	CL-CTX-CT-FEP-OT-S	3 (1.86) (ND in CS)	0.170
CS	6	4	CL-CT-FOX-N-OT-S	1 (0.62) (ND in BM)	0.132
CS	6	4	CT-FOX-ENR-OT-S-VA	1 (0.62) (ND in BM)	0.132
CS	7	4	AML-CL-CT-CTX-FEP-S-VA	1 (0.62) (ND in BM)	0.132
BM	7	4	CL-CT-CTX-FEP-FOX-OT-S	2 (1.24) (ND in CS)	0.294
BM	7	4	CL-CT-DO-FEP-FOX-OT-S	1 (0.62) (ND in CS)	0.485
CS	7	4	CT-CTX-FEP-FOX-N-OT-S	1 (0.62) (ND in BM)	0.132
CS	8	4	AML-AMP-CL-CT-CTX-E-FOX-S	1 (0.62) (ND in BM)	0.132
CS	8	4	CL-CT-CTX-DO-FEP-FOX-OT-S	1 (0.62) (ND in BM)	0.132
BM	9	4	AML-CT-CTX-DO-E-FEP-FOX-OT-S	1 (0.62) (ND in CS)	0.485
CSBM	9	4	CL-CT-CTX-ENR-FEP-FOX-N-S-VA	1 (0.62)	0.485
	9	4	CL-CT-CTX-ENR-FEP-FOX-N-S-VA	1 (0.62)	
CS	5	5	CT-E-OT-S-SXT	1 (0.62) (ND in BM)	0.132
CS	7	5	CL-CT-E-FOX-FEP-OT-SXT	1 (0.62) (ND in BM)	0.132
CS	7	5	CL-CT-CTX-FEP-OT-S-SXT	1 (0.62) (ND in BM)	0.132
CS	7	5	CL-CT-CTX-E-FOX-OT-S	1 (0.62) (ND in BM)	0.132
BM	7	5	CL-CT-CTX-ENR-S-SXT-VA	1 (0.62) (ND in CS)	0.485
CS	8	5	CL-CT-CTX-E-FEP-FOX-OT-S	1 (0.62) (ND in BM)	0.132
CS	9	5	AML-CL-CT-ENR-FEP-OT-S-SXT-VA	1 (0.62) (ND in BM)	0.132
CS	9	5	CL-CT-CTX-ENR-FEP-FOX-S-SXT-VA	1 (0.62) (ND in BM)	0.132
CS	11	5	AML-AMP-C-CL-CT-CTX-E-ENR-FEP-FOX-VA	1 (0.62) (ND in BM)	0.132
CS	8	6	CL-CT-E-FOX-N-OT-S-SXT	1 (0.62) (ND in BM)	0.132
CS	9	6	CL-CT-CTX-E-FEP-FOX-OT-S-SXT	1 (0.62) (ND in BM)	0.132
CS	9	6	CL-CT-CTX-E-FOX-N-OT-S-SXT	1 (0.62) (ND in BM)	0.132
BM	9	6	C-CL-CT-E-FEP-FOX-N-OT-S	1 (0.62) (ND in CS)	0.485
CS	10	6	AML-CL-CT-CTX-E-ENR-FEP-N-OT-S	1 (0.62) (ND in BM)	0.132
CS	10	6	CL-CT-DO-E-ENR-FEP-FOX-N-S-VA	1 (0.62) (ND in BM)	0.132
CS	12	6	CL-CT-CTX-DO-E-ENR-FEP-FOX-N-OT-S-VA	1 (0.62) (ND in BM)	0.132
CS	8	7	CL-CT- DO-E-ENR-OT-S-SXT	1 (0.62) (ND in BM)	0.132
CS	10	7	CL-CT-CTX-E-ENR-FEP-FOX-OT-S-SXT	1 (0.62)(ND in BM)	0.132
Total MDRE^5^ isolates from cloacal swab samples				61 (37.89)	0.023*
Total MDRE isolates from breast meat samples				44 (27.33)	

AML=Amoxicillin, AMP=Ampicillin, C=Chloramphenicol, CL=Cephalexin, CT=Colistin, CTX=Cefotaxime, DO=Doxycycline, E=Erythromycin, ENR=Enrofloxacin, FEP=Cefepime, FOX=Cefoxitin, N=Neomycin, OT=Oxytetracycline, S=Streptomycin, SXT=Sulfamethoxazole+trimethoprim, VA=Vancomycin. ^1^AMR=Antimicrobial resistance. ^2^AM=Antimicrobial. ^3^The statistical significance level was set at 95% confidence. The AMR profile was compared if *Enterococcus* was detected in both cloacal swab and breast meat samples using the Chi-square test. ^4^ND=Not detected. ^5^Multidrug-resistant *Enterococcus*.

* Prevalence of AMR profile between cloacal swab and breast meat samples was significantly difference (p *<* 0.05).

Subsequently, 51 of the 105 MDRE isolates sampled from cloacal swabs and breast meat samples of all 31 slaughterhouses were tested for 5 AMRGs (Table-4). The selected phenotypic MDRE isolates showed resistance to beta-lactam, CL, and VA antibiotics, associated with genotypic ESBL-Temoneira (TEM), *bla*_TEM_, *mrc–1*, and *vanA* resistance genes. Besides, the highest AMRG found in both cloacal swab and breast meat samples was ESBL–TEM, at prevalence rates of 71.43% (20/28) and 78.26% (18/23), respectively, and the other three AMRGs detected in the cloacal swab samples were *intI1, vanA*, and *mrc–1*, at prevalence rates of 14.28% (4/28), 14.28% (4/28), and 3.57% (1/28), respectively. Results also showed that although the only isolate from breast meat samples contained *bla*_TEM_ (1.96%), *intI1*, *mrc–1*, and *vanA* were absent from the breast meat samples.

**Table-4:** Detection of the five AMRGs^1^ of selected phenotypic MDRE^2^ isolates from slaughterhouses in Nakhon Ratchasima Province, Thailand.

Sample	Percentage of selected MDRE isolates associated with AMRGs				

	ESBL-TEM	*bla*_TEM_	*intI1*	*mrc–1*	*vanA*
Cloacal swab	71.43 (20/28)	0 (0/28)	14.28 (4/28)	3.57 (1/28)	14.28 (4/28)
Breast meat	78.26 (18/23)	4.35 (1/23)	0 (0/23)	0 (0/23)	0 (0/23)

^1^AMRGs=Antimicrobial resistance genes. ^2^MDRE=Multidrug-resistant *Enterococcus.* ESBL–TEM=Extended-spectrum beta-lactamase–Temoneira

## Discussion

The prevalence of *Enterococcus* isolates has been reported in humans, animals, and the environment. Specifically, most *Enterococcus* isolates in humans are observed in patients admitted to hospitals, whereas those in animals are in poultry animals and products. In contrast, those in the environment are related to farming food-producing animals and hospital environments. Therefore, based on this study’s background, the prevalence rate of *Enterococcus* isolates in cloacal swabs and meat samples of chickens at all local poultry slaughterhouses in Nakhon Ratchasima Province was investigated and their prevalence rates were 29.75% and 27.95%, respectively. The previous study reported that the prevalence of *Enterococcus* spp. in broiler meat and water used for broiler farming in Thailand to be 25.1% and 17.2%, respectively [15]. However, the prevalence of *Enterococcus* isolates in chicken feces from six provinces in three Thai regions was reported to be 18.67% [16]. These results presented a lower prevalence rate than we found in our study (29.75%), which may be because the study reported only two *Enterococcus* species, *E*. *faecalis* and *E*. *faecium*. Additionally, a study from Southeast Asian countries (Thailand, Vietnam, and Indonesia) reported high prevalence rates of *Enterococcus* isolates in chicken feces (86.34%) [17] which may be due to contamination of the environment via wastewater from both hospital and animal farm wastes [18, 19]. Moreover, our study sampled various chicken types (indigenous, broilers, and laying hens) and yielded different results.

In this study, *Enterococcus* isolates from chicken meat showed higher CL and FOX resistance than those from chicken feces but were not significantly different (p = 0.814, p = 0.628), suggesting that the increasing resistance of *Enterococcus* isolates came from increased environmental contamination during slaughtering. This result was similar to that of de Jong *et al*. [20]. Resistance to this study’s third and fourth generations of beta-lactam cephalosporin antibiotics (CTX and FEP) indicates an MDR trend in *Enterococcus* isolates, which agrees with recent studies concerning antibiotic-resistant bacteria worldwide. Furthermore, we observed a high prevalence rate of ESBL-TEM-resistant genes from both chickens (71.73%) and chicken meat (78.26%) of the *Enterococcus* isolates (Table-4), supporting the high MDRE isolates in this study.

First to third-generation cephalosporins have been used as a drug of choice to treat Gram-negative bacterial infections, such as human salmonellosis, for >30 years. However, cephalosporin resistance can come from overuse/misuse in Thai food-producing animals [21]. Additionally, cephalosporins can produce mutant enzymes, such as TEM or sulfhydryl variable (SHV), through their plasmids [22]. *Enterococcus* has a natural resistance to beta-lactam, which possesses low-affinity penicillin-binding proteins and low-level aminoglycoside antibiotics. However, acquired resistance to beta-lactam through penicillinase production and high-level aminoglycoside (gentamicin) production has been reported [23]. Extended-spectrum cephalosporins (e.g., at least third generation) lead to acquired resistance mediated by AmpC beta-lactamases and ESBL-encoding genes (e.g., TEM, SHV derivative, and CTX-M family), have also been reported in Gram-negative pathogens [12, 24]. However, little is known about commensal Gram-positive bacteria, such as enterococci. Our study showed that the prevalence of MDRE resistance to at least one of the third (CTX) and fourth-generation (FEP) cephalosporins was 50.48% (53/105). Additionally, we observed a positive ESBL-TEM gene from phenotypic MDRE at a prevalence rate of 74.51% (38/51). This result indicated that the positive correlation between MDR with CTX/FEP-resistant *Enterococcus* isolates and acquired ESBL-TEM-resistant genes in enterococci leads them to act as resistant gene reservoirs for bacterial transfer.

Alternatively, although CT use in Thai animal feeds has only been allowed for short-term treatment and has been prohibited in animal feeds since 2019 [25], it is continued to be used to treat severe Gram-negative bacterial infections in humans, such as carbapenemase-producing *Enterobacteriaceae*. International organizations such as FAO and CODEX have recently been concerned with incorporating the *mcr–1* gene (a Gram-negative bacterial gene) into human and veterinary disease treatments to make them pan-drug resistant, as reviewed by Gharaibeh and Shatnawi [26]. Here, we observed that although most *Enterococcus* isolates showed intrinsic resistance to CT with a prevalence of 98.1% (158/161), one *Enterococcus* isolate had the *mcr–1* gene, serving as a gene reservoir for bacterial transfer.

The prevalence rate of S resistant *Enterococcus* (SRE) isolates in this study (60.1%) was consistent with that recorded in other studies, which reported the prevalence rate SRE isolated from broiler chicken feces as 63.5%–69.5% in Taiwan [27] and 78.6% in Vietnam [17]. These findings also support the natural resistance of *Enterococcus* spp. to aminoglycosides, similar to the reports of previous studies [28, 29]. However, our study was inconsistent with one report, according to which 25.22% of S-resistant *Enterococcus* isolates were identified in chicken feces in six Thai provinces [16].

In this study, the prevalence of MDRE isolates resistant to several antibiotics (3–12) was 65.22% (105/161). Our results agrees with those of Desire *et al*. [30], who reported an MDRE prevalence in laying hens of 73.17% (30/40), with the number of antibiotics ranging from three to eight. Furthermore, compared with those in the aforementioned study, we observed that various MDRE patterns in our study were more significant (66 vs. 32 patterns), confirming the possibility of *Enterococcus* spp. as AMR reservoirs for bacterial transfer.

Vancomycin-resistant enterococci, particularly *E*. *faecium*, cause dominant nosocomial infections in humans [31]. It has also been reported that animal farming can cause resistance of enterococci to vancomycin due to the use of avoparcin as an antibiotic or for growth promotion. Avoparcin, a glycopeptide analog of VA, contributes to the high prevalence of VRE in food animals; thus, its resistant genes can be transferred to humans. Although the EU (completely banned in 2006) and many countries worldwide have banned antimicrobial growth promoters, the persistence of VRE in animal husbandry can still be detected because of its long-term viability. Additionally, VRE can be transferred to hospitalized patients or hospital environments through animal wastewater [32]. Moreover, transferable VRE genes from humans to poultry through transposons have been reported by van den Bogaard *et al*. [33]. The *vanA* gene could, in turn, transfer most genes from poultry to human enterococci [34]. Alternatively, the prevalence rates of VRE isolates have been reported heterogeneously from country to country, including in Italy, where the *Enterococcus* isolates from humans, cats, and dogs feces resistant to VA were 0%, 23.6%, and 6.2%, respectively [35]. In Ethiopia, the pooled prevalence of VRE isolated from patients in hospitals was 16.9% [36], whereas the prevalence of those from broiler chicken feces sampled between 2000 and 2003 in Taiwan was 1.85–8.55% [27]. Additionally, the prevalence rate of VRE has also been reported in hospital wastewater samples, such as 36% in Sweden [37]. Since the Thai government banned the use of avoparcin in animal feed in July 1998, studies have reported low prevalence rates ranging from 0% to 10.3% of VRE in Thai poultry animals [15, 16], similar to those recorded in this study. Although this study recorded a phenotypic VRE prevalence of 15.66% (13/83) and 7.69% (6/78) in fecal and meat samples, respectively, only 4 of 28 phenotypic MRDE isolates from chicken feces were positive for the *vanA* gene, indicating a low risk of *vanA* emergence in chicken meat. This may be due to all studied samples were collected from poultry farms and slaughterhouses that follow the minimum requirements for good hygienic systems, such as Good Agriculture Practices and Good Manufacturing Practices for poultry farming and poultry slaughterhouses [38], respectively. Additionally, these farms and slaughterhouses had been certified by the Department of Livestock Development, Thailand. Nevertheless, these systems may not be completely adequate for biosafety and biosecurity application to prevent contaminated commensal bacteria, which have potential pathogens such as MRDE strains. Therefore, we suggest the use of thorough hygienic practices, for instance, Hazard Analysis Critical Control Point in these poultry slaughterhouses in Nakhon Ratchasima Province, Thailand.

## Conclusion

This study indicated that chicken meat from local poultry slaughterhouses in Nakhon Ratchasima Province, Thailand, had low VRE infection risks. However, the high prevalence of MDRE isolates in this study remains a public health concern. Hence, AMR surveillance in poultry meat production should be considered, including adopting effective biosafety and biosecurity systems for animal food products, such as good sanitation for poultry farming and slaughtering.

## Data Availability

Supplementary data can be available from the corresponding author on a reasonable request.

## Authors’ Contributions

PS and PN: Conceptualized and designed the study. PN, CN, TS, and SL: Contributed to sample collection, microbiological culturing, and PCR running. PS and PN: Performed statistical analyses. PN: Drafted the manuscript. PS: Revised the manuscript. All authors have read and approved the final manuscript.
